# An implantable cardioverter defibrillator in a patient with triple mechanical valves

**DOI:** 10.1002/ccr3.1721

**Published:** 2018-07-23

**Authors:** Alex W. X. Tan, Kah L. Ho, Chi K. Ching, Kelvin C. M. Chua

**Affiliations:** ^1^ National Heart Centre Singapore Singapore Singapore

**Keywords:** cardiovascular disorders, complete heart block, implantable cardioverter‐defibrillator lead implantation in the coronary sinus, triple mechanical valves

## Abstract

A patient with permanent atrial fibrillation, triple mechanical prosthetic valve replacements, and nonischemic cardiomyopathy presented with symptomatic high‐grade atrioventricular block. A transvenous implantable cardioverter‐defibrillator system was achieved with the defibrillator lead and bipolar pace‐sense lead in separate anterolateral branches of the coronary sinus with successful defibrillation testing.

## 1 CASE REPORT

We present a 67‐year‐old man with permanent atrial fibrillation and triple mechanical prosthetic valve replacements (All bileaflet mechanical valves, St Jude Medical Inc, St Paul, MN, USA) at the aortic, mitral, and tricuspid positions since 1999 for rheumatic valvular disease. He subsequently developed progressive nonischemic cardiomyopathy with a residual left ventricular ejection fraction of 15% for which a subcutaneous implantable cardioverter defibrillator (S‐ICD) was recommended but he declined.

**Figure 1:**
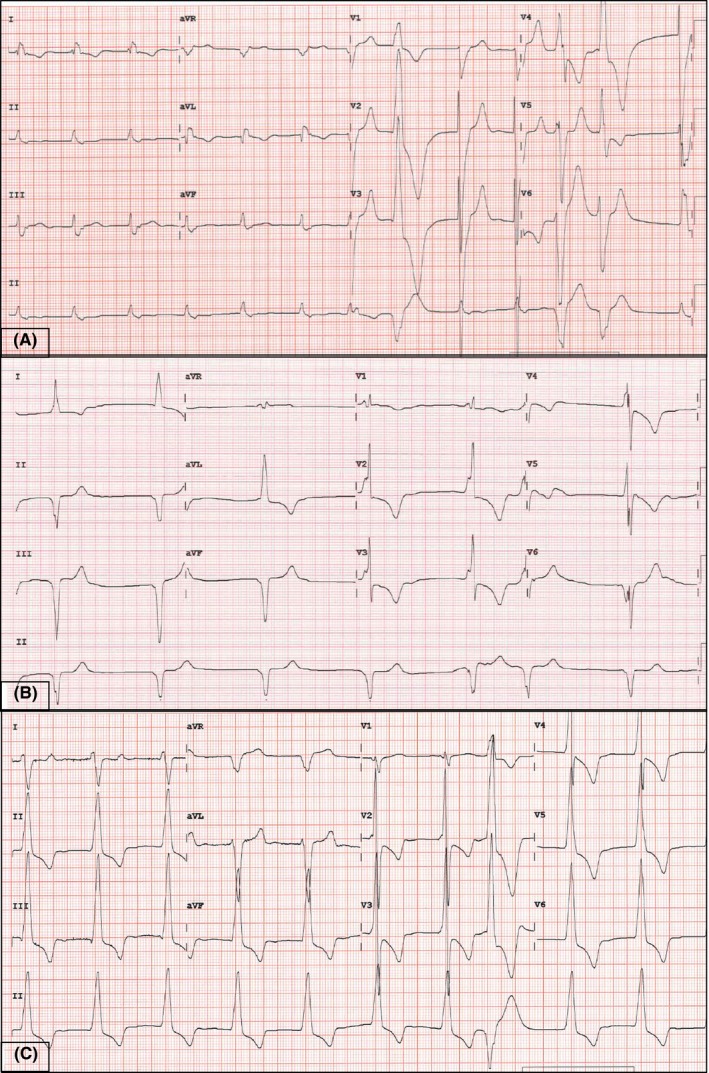
A, Baseline electrocardiogram (ECG) showing atrial fibrillation (AF) with left bundle branch block (LBBB) morphology and premature ventricular complexes (PVC) of right bundle branch block (RBBB) and superior axis morphology; B, Presenting ECG showing AF with intermittent rate regularization and slight variation in wide complex QRS morphology suggestive of complete heart block with variable ventricular escape rhythm. C, Postprocedural ECG showing basal anterolateral pacing with a similar PVC of RBBB and superior axis morphology

He presented to the emergency department in October 2017 for increasing dyspnea and orthopnea. Physical examination revealed significant bradycardia of 35‐40 bpm with signs of congestive cardiac failure. An electrocardiogram (ECG) performed showed atrial fibrillation with intermittent rate regularization and variable wide complex QRS morphology (Figure 1A,B), which was suggestive of high‐grade atrioventricular (AV) block with variable ventricular escape rhythm. We counseled the patient and scheduled for an urgent cardiac resynchronization therapy with defibrillator (CRT‐D) device implantation via the coronary sinus.

**Figure 2:**
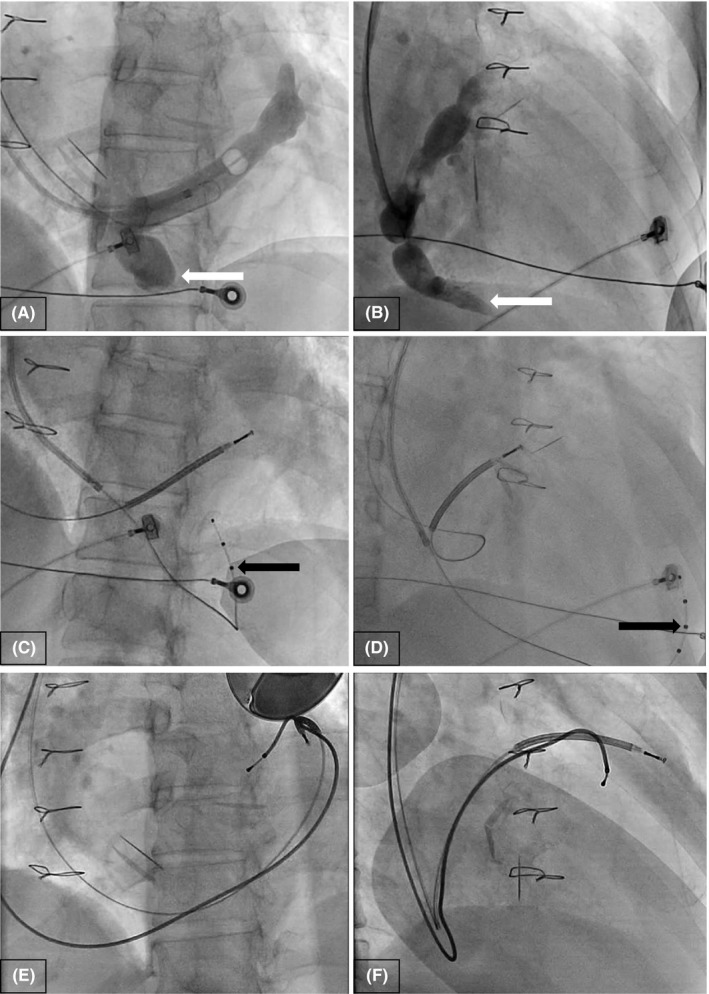
A, B, Occlusive coronary sinus (CS) venography in left anterior oblique (LAO) and right anterior oblique (RAO) projection and showing an ectatic CS with a suggestion of posterolateral and anterolateral branches and also a middle cardiac vein (MCV) with an ectatic proximal segment (white arrow). C, D, Fluoroscopic LAO and RAO projections showing the defibrillator lead in the main CS body and quadripolar lead deep in the MCV wrapping around the apex to the anterior interventricular vein (AIV) (black arrow). E, F, Fluoroscopic LAO and RAO projections showing defibrillator lead and bipolar pace‐sense lead in anterolateral branches of CS as well as triple mechanical valve prostheses

Under aseptic conditions, extrathoracic subclavian venous punctures were made using fluoroscopic guidance. The guidewires were easily maneuvered into a large coronary sinus (CS). An occlusive CS‐venogram demonstrated an ectatic CS with tortuous takeoffs at the posterolateral branches. The middle cardiac vein (MCV) had a separate ostium which was also ectatic at its proximal segment tapering into a much smaller vessel (Figure 2A,B).

Using an 0.014″ guidewire (Acuity Whisper View EDS 190 cm, Boston Scientific Corp, St Paul, MN, USA) through an extended‐hook outer sheath (Acuity Pro 9F Guiding Catheter Extended Hook 45 cm, Boston Scientific Corp) and inner catheter (Acuity Pro 7F Inner Guiding Catheter CS‐IC130 60 cm, Boston Scientific Corp), we were able to advance the wire into the MCV after numerous attempts. However, the outer sheath could not follow through distally, and even if it did, it would not accommodate the larger caliber defibrillator lead. Hence, we proceeded to advance a straight quadripolar lead (Acuity X4 straight 86 cm, Boston Scientific Corp) into the MCV, going around to the anterior interventricular vein (AIV) to achieve a stable position. (Figure 2C,D) We then directed a defibrillator lead (Endotak Reliance SG 64 cm DF4, Boston Scientific Corp) into the CS just using a curved stylet and advanced it into an anterolateral branch for stability without helix deployment. During interrogation of the defibrillator lead, however, we found that there were poor R‐wave sensing and unacceptably high thresholds and we could not use the quadripolar lead in the MCV for tachycardia sensing.

Hence, we removed the quadripolar lead in the MCV and advanced a bipolar lead (Acuity Steerable 90 cm IS1, Boston Scientific Corp) into the MCV, but we could not advance it to the AIV due to an increase lead caliber. Hence, we advanced it to an anterolateral branch of the CS. We also successfully switched the defibrillator lead to one with a DF1 connection (Endotak Reliance SG 64 cm DF1, Boston Scientific Corp) and readvanced it into another anterolateral branch of the CS. (Figure 2E,F) The leads were then connected to a generator (Inogen EL ICD, Boston Scientific Corp) with the atrial port plugged.

We then proceeded with defibrillation testing. We made five attempts at induction of ventricular tachyarrhythmia using shock‐on‐T as well as 50 Hz burst pacing. For three attempts, we did not induce any tachyarrhythmia. For two other attempts, we induced a rapid monomorphic ventricular tachycardia (MMVT) that was nonsustained. We decided not to persist with defibrillation testing. Postprocedure device check showed satisfactory R waves of 14 mV, a pacing threshold of 0.8 V at 0.4 ms, and stable impedance of 765 ohms. A 12‐lead ECG pacing pattern was consistent with baso‐anterolateral LV pacing. (Figure 1C).

**Figure 3:**
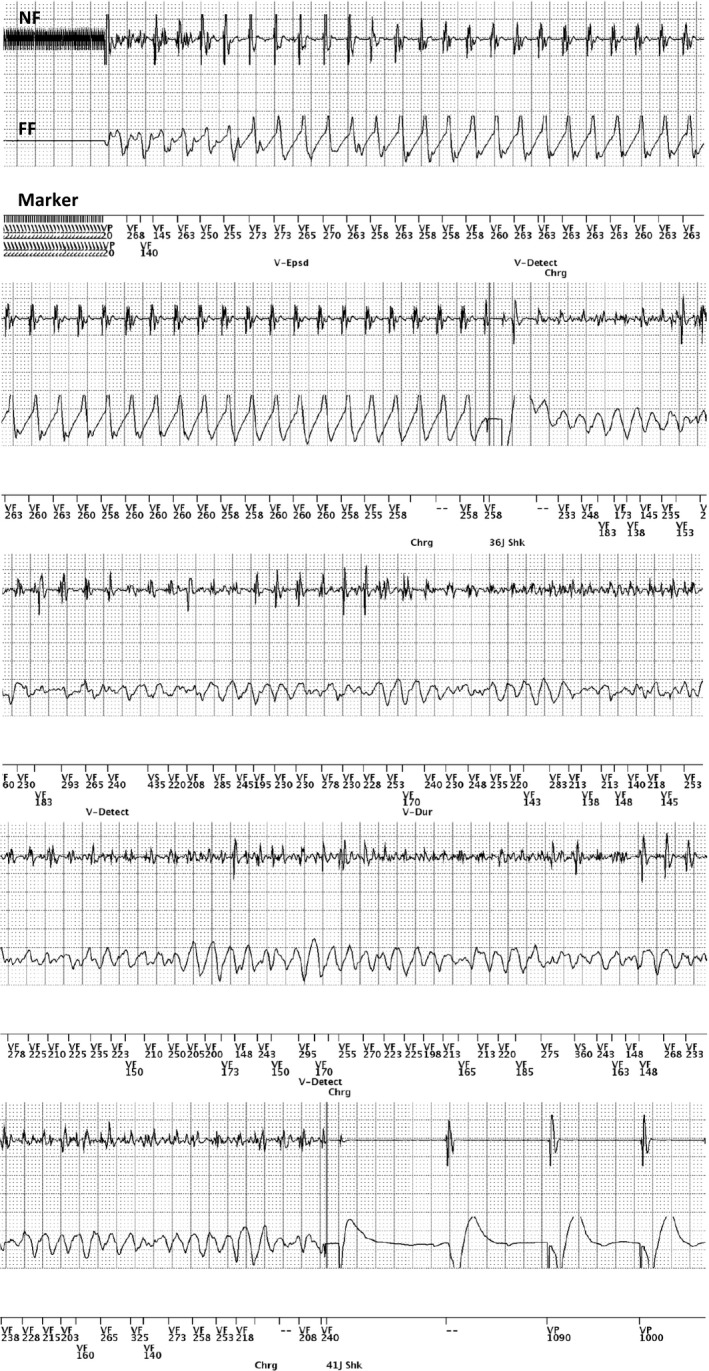
Device electrograms (EGM) showing induction of monomorphic ventricular tachycardia using 50 Hz burst pacing, appropriate detection with no undersensing, appropriate charge time, and delivery of 36J shock which degenerates the rhythm into ventricular fibrillation. This is redetected appropriately, and a delivery of 41J shock successfully cardioverts the patient back to baseline paced rhythm

The patient was reviewed in the outpatient clinic 1 month later, and device interrogation showed stable parameters with recorded episodes of nonsustained ventricular tachycardia (NSVT). The patient agreed to repeat defibrillation testing and was readmitted for this. After moderate sedation, rapid MMVT was induced with 50 Hz burst pacing through the device, and an initial programmed defibrillation at 26J degenerated the rhythm into ventricular fibrillation (VF), while the next programmed defibrillation at 41J successfully cardioverted the patient back to ventricular paced rhythm. We reinduced MMVT with 50 Hz burst pacing, and again the first programmed defibrillation at 36J degenerated the rhythm into VF, while the next programmed defibrillation at 41J successfully cardioverted the patient back to ventricular paced rhythm. (Figure 3) A long discussion was made with the patient and family, and a decision was made not to pursue with further lead repositionings nor additional defibrillator coils or arrays in a bid to reduce the defibrillation threshold.

## 2 DISCUSSION

Placement of a transvenous right ventricular endocardial pacing wire through a mechanical tricuspid valve is considered a contraindication although there was reported success previously success.^1^ The issues with this approach include prosthetic valve damage, failure, and lead malfunction. Retrospective data suggest that patients who undergo tricuspid valve surgery have an increased risk of conduction disorders requiring cardiac implantable electronic devices, and this risk is doubled with multivalve surgery.^2^ In this regard, some centers advocate prophylactic placement of permanent epicardial leads in high‐risk patients at the time of surgery. However, epicardial leads may have an increased risk of lead failure and higher thresholds necessitating revisions and frequent generator replacements compared to transvenous systems and this would mean repeat thoracotomies.^3^

Our patient had indications for cardiac resynchronization, but we only fulfilled the pacing and defibrillation requirements. The other options we considered were a subcutaneous ICD with a leadless pacemaker but that would involve a large sheath temporarily across the mechanical valve which may risk valve damage.^4^ We considered a subcutaneous ICD with a transvenous pacing system in a branch of the CS, but this would involve two incisions with an increased risk of infection and uncertain interactions between the two systems. We also contemplated a completely epicardial system, but the surgical team was reluctant to proceed with a repeat thoracotomy, and furthermore, epicardial systems may have limited longevity as discussed above.

Transvenous ICD systems in patients with mechanical tricuspid valves have been achieved in several ways including the use of a floating double‐coil in the inferior vena cava (IVC), azygous vein ICD lead implant, the use of a CS defibrillation coil coupled with a left‐sided array, and placement of the ICD lead in the low right atrium or MCV.^5^, ^6^, ^7^, ^8^, ^9^ In our patient, the MCV was tortuous, tapering into a small caliber vessel proximally, and we did not have appropriately sized sheaths to assist in the delivery of the ICD lead into the MCV. There have been reports of using more specialized sheaths or smaller ICD leads, but we did not have the luxury of these options.^9^, ^10^ Importantly, we did not deploy the ICD lead helix as sensing was already provided by the bipolar pace‐sense lead and we wanted to minimize the risk of CS trauma and perforation.

We avoided the need for general anesthesia and a repeat thoracotomy in our patient and perhaps mitigated the risks associated with it. However, we were not able to induce a sustained tachyarrhythmia during the procedure for defibrillation testing. When the patient was brought back for defibrillation testing, we were unable to demonstrate any defibrillation safety margin.

The management of a patient with complete heart block, nonischemic cardiomyopathy and mechanical tricuspid valve replacement remains challenging especially in terms of anatomical constraints and high defibrillation thresholds. This case highlights the important considerations in determining the most appropriate device system for such patients and underscores the technical challenges to ensure a safe and successful implantation.

## CONFLICT OF INTEREST

None declared.

## AUTHORSHIP

AT: conceived and drafted the manuscript. KLH and CKC: contributed to procedural assistance and proofreading of the manuscript. KCMC: contributed to concept, collation of the data, editing figures, and proofreading of the manuscript.
